# Inhibition of mTOR signaling by genetic removal of p70 S6 kinase 1 increases anxiety-like behavior in mice

**DOI:** 10.1038/s41398-020-01187-5

**Published:** 2021-03-15

**Authors:** Muriel Koehl, Elodie Ladevèze, Caterina Catania, Daniela Cota, Djoher Nora Abrous

**Affiliations:** 1 Univ. Bordeaux, INSERM, Neurocentre Magendie, U1215, Neurogenesis and Pathophysiology Group, F-3300 Bordeaux, France; 2 Univ. Bordeaux, INSERM, Neurocentre Magendie, U1215, Energy Balance and Obesity Group, F-3300 Bordeaux, France

**Keywords:** Neuroscience, Physiology

## Abstract

The mechanistic target of rapamycin (mTOR) is a ubiquitously expressed kinase that acts through two complexes, mTORC1 and mTORC2, to regulate protein homeostasis, as well as long lasting forms of synaptic and behavioral plasticity. Alteration of the mTOR pathway is classically involved in neurodegenerative disorders, and it has been linked to dysregulation of cognitive functions and affective states. However, information concerning the specific involvement of the p70 S6 kinase 1 (S6K1), a downstream target of the mTORC1 pathway, in learning and memory processes and in the regulation of affective states remains scant. To fill this gap, we exposed adult male mice lacking S6K1 to a battery of behavioral tests aimed at measuring their learning and memory capabilities by evaluating reference memory and flexibility with the Morris water maze, and associative memory using the contextual fear conditioning task. We also studied their anxiety-like and depression-like behaviors by, respectively, performing elevated plus maze, open field, light-dark emergence tests, and sucrose preference and forced swim tests. We found that deleting S6K1 leads to a robust anxious phenotype concomitant with associative learning deficits; these symptoms are associated with a reduction of adult neurogenesis and neuronal atrophy in the hippocampus. Collectively, these results provide grounds for the understanding of anxiety reports after treatments with mTOR inhibitors and will be critical for developing novel compounds targeting anxiety.

## Introduction

The mechanistic (or mammalian) target of rapamycin (mTOR) is an evolutionarily conserved serine/threonine protein kinase that plays a key role in regulating protein synthesis. The mTOR pathway integrates signals from nutrients, growth factors, and energy status to regulate many processes, including cell growth, proliferation, motility, and survival^1,2^. In neurons, the mTOR pathway modulates local translation of proteins at the synapse and therefore is critical for different forms of synaptic plasticity^3,4^. Taken together with its ubiquitous expression, it is thus not surprising that dysfunction of mTOR signaling represents a common hallmark in a wide variety of brain disorders, including autism, tuberous sclerosis, neurofibromatosis, fragile X or Rett syndrome, and neurodegenerative disorders, such as Parkinson’s, Alzheimer’s, or Huntington’s disease^5^.

mTOR, therefore, constitutes an attractive therapeutic target, and great effort has been made to determine its therapeutic indications. For instance, mTOR inhibitors such as rapamycin or its analog everolimus are now approved for treating various disorders including cancer, and as immunosuppressive drugs in solid organ transplantation. Furthermore, many preclinical and clinical studies are under way to test the efficiency and safety of mTOR inhibition in cystic diseases, neurodegenerative diseases, or metabolic disorders^6^. However, the signaling pathways that are regulated by mTOR are complex and a considerable number of metabolic or physiological side effects have been described after treatments with inhibitors^6^. In particular, and consistent with the involvement of the mTOR pathway in synaptic plasticity and memory processing^7^, treatment with these inhibitors was found to affect cognition and affective states, with highly discrepant results.

On one hand, rapamycin treatment in humans and preclinical animal models may induce significant cognitive impairment^8,9^ and increase depressive-like and anxiety-like behavior^10–12^. The latter observation is consistent with mouse models of disorders that impact mTOR signaling in which abnormal anxiety-like behaviors have frequently been demonstrated^13,14^. On the other hand, everolimus treatment of heart transplant patients previously treated with calcineurin inhibitors has been associated with significant improvements in memory and concentration functions and in mood and quality of life, as well as global psychiatric symptoms, indicating a positive effect of everolimus, although a spontaneous recovery from the deleterious side effects of calcineurin inhibitors cannot be excluded^15^. In agreement with this clinical dataset, rapamycin or everolimus treatment in adult mice was found to improve spatial learning and memory capabilities and decrease depressive-like and anxiety-like behaviors^16,17^. Finally, other studies also reported that everolimus treatment did not affect learning and memory, and had no influence on depression-like or anxiety-like behavior^18^.

Altogether, these data indicate that there is no clear correlation between activity of mTOR pathway and side effects such as cognitive deficits, anxiety or depression. Such discrepancy could be linked to the complexity and broadness of the mTOR network/signaling^19^. Indeed, mTOR acts in cells by forming two distinct complexes, called mTOR complex 1 (mTORC1) and mTOR complex 2 (mTORC2). mTORC1 functions as a nutrient/energy/redox sensor; its effects are mediated by the phosphorylation of downstream proteins, such as the 70-kDa ribosomal protein S6 kinase 1 (S6K1), which in turn controls protein homeostasis. While mTORC2 activates Akt/protein kinase B, which plays a central role in the control of cell metabolism, cell stress resistance and cytoskeleton regulation. As treatments with the mTOR inhibitors can affect one, the other, or both complexes^20^, different resulting effects can be expected. The development of new, more specific therapeutic tools to prevent these deleterious behavioral side effects thus depends on a better characterization of the involvement of the different effectors of the pathway in regulating cognition and affective states.

We therefore investigated the consequences of blocking the mTORC1 pathway on cognitive and emotional behavior, as well as on adult hippocampal neurogenesis as a potential mechanistic substrate of the behavioral measures, using genetically-engineered mice deficient for S6K1^21^. This mouse model has been extensively used in the metabolism and aging field (see for example refs. ^22–25^), but only few studies have investigated it in the context of CNS-related functions. Of these, it was shown that genetic deletion of S6K1 does not rescue phenotypic deficiencies observed in a mouse model of Huntington’s disease^26^, and does not mediate PTEN-deficient neuronal hypertrophy^27^. Using this same model, we have shown that S6K1-KO mice are characterized by decreased hypothalamic neuroinflammation^28^, and that they do not respond to the appetite-suppressant action of diverse factors^21,29^. To the best of our knowledge, only one study investigated the involvement of mTORC1 pathway in cognitive and emotional behavioral outputs using this model, and reported that adult S6K1-KO mice exhibit deficits in memory acquisition visible in contextual fear memory and conditioned taste aversion tests but do not display differences in anxiety-like behavior^30^.

## Material and methods

### Animals

**Fig. 1:**
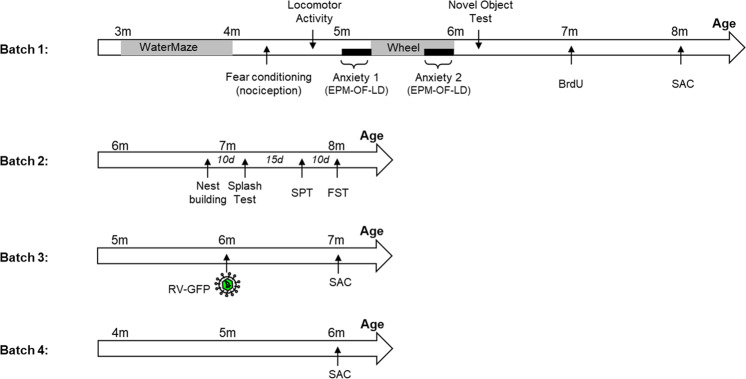
Timeline of the experiments. Mice from 4 batches were used to assess the impact of S6K1 removal on learning and memory abilities along with anxiety-related behavior (batch 1); on depression-like behavior (batch 2); on dendritic morphology of adult-born granule neurons (batch 3); on dendritic morphology of all granule neurons (batch 4).

Male S6K1^−/−^ mice (henceforth named S6K1-KO) and their WT littermates were obtained and genotyped as described^28^. At eight weeks of age, animals were housed individually in standard plastic rodent cages and maintained on a 12 h light/dark cycle (light on at 7 a.m.) with free access to water and food. Four batches of mice were used (Fig. 1): Batch 1 (*n* = 11 mice/genotype) was used for characterizing the impact of S6K1 deletion on memory abilities, anxiety-related behavior, exploratory behavior, and adult neurogenesis; Batch 2 (*n* = 6 mice/genotype) was used to characterize depression-related behavior; Batch 3 (*n* = 4 WT and *n* = 6 S6K1-KO mice) was used to analyze the dendritic morphology of newborn dentate granule neurons; and Batch 4 (*n* = 3 mice/genotype) was used to assess the dendritic morphology of all dentate granule neurons by Golgi staining. Sample size was chosen based on our previous experience with these tests. For all behavioral tests, testing order was randomized across genotypes and investigators were blinded to the genotype of mice; however because S6K1-KO mice are visually smaller in size, behavioral data analysis was performed offline by a third party who was also blinded to the group allocation. All sections were coded for immunohistological and dendrites measures and the codes were only broken at the end of the analysis. All experimental procedures have been carried out following the European directives of September 22, 2010 (2010/63/UE) and animal studies were approved by the ethical committee of Bordeaux (CEEA50; Dir 13105).

### General procedures

#### Measurement of memory abilities

##### Water maze

The apparatus was a white circular pool (150 cm in diameter) located in a room with various distal cues, and filled with water maintained at 20 °C and made opaque by the addition of a non-toxic white cosmetic adjuvant. Data were collected using a video camera fixed to the ceiling of the room and connected to a computerized tracking system (Videotrack, Viewpoint) located in an adjacent room. The tracking system allowed the calculation of escape latency and path length.

*Pre-training:* Mice (batch 1) received a three-step pre-training session. First, they were allowed to swim for 60 s in the water maze without the platform. Then, they were placed upon the platform (16 cm diameter) raised at the surface of the water where they were required to stay for at least 15 s. Finally, they were allowed to swim for a 30 s period that was ended by a climbing trial onto the hidden platform (1.5 cm below water level). At the end of the pre-training, all mice swam actively and were able to climb onto the platform and stay on it for 15 sec.

*Training with variable start positions:* Mice were required to locate the hidden platform using distal extra-maze cues. They received 3 daily trials separated by a 5 min inter-trial interval during which they were held in their home cages. A trial terminated when the animal climbed onto the platform or after a 60 s cut-off time. The starting point differed for each trial and different sequences of starting points were used day to day.

*Training with constant start positions:* Upon completion of the first training, platform location was changed to a different quadrant, and mice were required to find the hidden platform using constant starting points. Procedures were similar to the ones used for training with variable start positions. When performances reached a stable level, animals were tested to locate the hidden platform from a novel start position (1 trial).

##### Contextual fear conditioning

Conditioning took place in a transparent Plexiglas box (30 × 24 × 22 cm high) with a floor made of 60 stainless steel rods (2 mm diameter, spaced 5 mm apart) connected to a shock generator (Imetronic, Bordeaux, France). The box was cleaned with 70% ethanol before each trial. Animals (batch 1) were submitted daily for 3 days to a 5 min contextual conditioning session during which they freely explored the apparatus for 3 min upon which one electric footshock (0.7 mA, 50 Hz, 2 s) was delivered. Mice were then free to explore the cage for two more minutes. Freezing behavior was scored over the first three minutes preceding shock delivery by an experimenter blind to the genotype of mice.

To exclude a distorted nociceptive sensory perception of electric shocks, mice were submitted to a shock sensitivity protocol and tested in the hot plate test. The first test was carried out in the same conditioning chamber. Each mouse was administered seven 1 s footshocks of increasing amplitude (from 0.1 to 0.7 mA) with an intertrial interval of 30 s. Two observers, blind to genotype, scored shock sensitivity based on three behavioral strategies: flinching, running/jumping and vocalizing. Scoring indicated the first shock intensity at which each reaction was detected. For the second test, which measures potential genotype-related differences in nociception, mice were placed in a Plexiglas box on the surface of a hot plate which was maintained successively at 49, 52, and 55 °C. The stimuli were presented using ascending order of intensity at 30-min intervals. Latency for the mouse to raise and lick its paw or jump up was recorded. Mice were removed from the hot plate to prevent tissue damage if they did not respond within 30 s.

#### Measurement of anxiety-related behaviors

*The elevated plus maze (EPM)* was conducted in a transparent Plexiglas apparatus with two open (45 × 5 cm) and two enclosed (45 × 5 × 17 cm) arms that extended from a common central squared platform (5 × 5 cm). The floor of the maze was covered with black makrolon and was elevated 116 cm above the floor. The test session began with the mouse individually placed on the center square facing an open arm. Animals (batch1) were allowed to freely explore the maze for 5 min (90 lux dim light). A camera connected to a computer was utilized to track the mouse path during the entire session (©VideoTrack, Viewpoint). Automatic path analysis measured time spent in and total number of entries into the open and closed arms. Standard measures of rodent anxiety were calculated: % time and % entry in the open arms compared to total time and total entries into any arm of the maze; in addition, total number of entries and total distance traveled in the open and closed arms were taken as a measure of activity/exploratory tendency in the EPM.

*The open-field test* was used one day later as an additional measure of anxious-like behavior, as well as to evaluate locomotor performance and exploratory activity. It consisted of an illuminated square arena of 50 × 50 cm closed by a wall of 50 cm high and made in white PVC (light ~700 lux). Mice were placed individually in a corner of the arena and their activity was recorded for 10 min using a videotracking system (©VideoTrack, Viewpoint). Time spent and distance traveled in each zone (corners, periphery and centerfield) were recorded and analyzed.

*The light/dark emergence test* was conducted in the same open-field containing a cylinder (10 cm deep, 6.5 cm in diameter, dark gray PVC) located length-wise along one wall, with the open end 10 cm from the corner. The day following open-field exposure, mice were placed into the cylinder and tested for 15 min under bright light conditions (1500 lux). Initial latency to emerge from the cylinder, defined as placement of all four paws into the open field, as well as total number of exits from the cylinder and total time spent inside the cylinder were analyzed.

#### Measurement of exploratory behavior

*Locomotor activity* (batch 1) was recorded from 2 to 4 pm under dim light (50 lux) in racks of 8 activity cages (18.2 cm × 12 cm × 22 cm) made of transparent Plexiglas and isolated from the surrounding environment. Each cage was equipped with two beams of infrared captors and infrared counts were computed via an electronic interface coupling each cage with an on-line computer (Imetronic, Bordeaux, France).

*The novel object test* was conducted in the open-field described previously. Mice (batch 1) were allowed to freely explore the empty open-field for 30 min (“*habituation*” condition). After this phase, they were temporarily placed back into their home cage while an object (8 cm in height and 7 cm in diameter) was placed in the center of the open-field. Then animals were placed back into the open-field, now containing the cup (“*novel object*” phase), and tested for an additional 30 min. The time spent exploring the center of the open-field (target zone) in the presence and in the absence of the cup was measured.

#### Exposure to running wheels

To test whether anxiety-related behavior was a stable trait consistently expressed even under enrichment conditions, we equipped the mice home cages with low profile wireless running wheels (Med Associates). All mice had free access to a wheel for 3 weeks and the number of wheel revolutions was recorded daily. Anxiety was tested before and at the end of the 3 weeks exposure (Fig. 1).

#### Measurements of depression-related behaviors

In a different batch of animals (Batch 2), the influence of S6K1 deletion on depression-related behaviors was examined by measuring *avolition* (lack of motivation or inability to initiate goal-directed behavior) in the nest building and sucrose splash tests, *anhedonia* in the sucrose preference test, and *resignation/behavioral despair* in the Forced swim test (FST)^31,32^.

##### Nest building

A cotton nestlet was placed in each cage in the morning and nest quality was evaluated 24 h later using the following criteria: Score 1: intact cotton square; Score 2: partially used cotton square; Score 3: scattered cotton; Score 4: cotton gathered in a flat nest; Score 5: cotton gathered into a “ball” with a small passage for entry of the animal.

##### Sucrose splash test

Ten days later, a high viscosity 10% sucrose solution was sprayed on the coat of the mice to induce a self-grooming behavior^33^. Latency to initiate the first grooming episode, as well as frequency and duration of grooming over a 5-min period was measured immediately after applying the solution.

##### Sucrose preference test

Two weeks later, mice were first habituated for 48 h to the presence of two drinking bottles filled with tap water. They were then given, for 48 h, a free choice between one bottle filled with a 4% sucrose solution, and the other with tap water. To prevent possible effects of side preference in drinking behavior, the position of the bottles was switched after 24 h. The consumption of water and sucrose solution was estimated by weighing the bottles. Sucrose intake was calculated as the amount of consumed sucrose in mg per gram body weight, and sucrose preference was calculated according to the formula: sucrose preference = (sucrose intake)/(sucrose intake + water intake) × 100.

*The Forced swim test (FST)* was performed ten days later by individually placing mice into a glass cylinder (height 25 cm; Ø 18 cm) filled with 26 °C water to a depth of 20 cm. Behavior was recorded for 6 min with a camera positioned to view the top of the cylinder. The latency to float and the duration of immobility were scored off-line by an experimenter unaware of the experimental groups. Only immobility scored in the last four minutes of the session was analyzed, and a mouse was judged to be immobile when it remained floating in an upright position, making only the movements necessary to keep its head above the water.

#### Thymidine analog injections

Animals from batch 1 were injected with Bromo-2’desoxyuridine (BrdU, 50 mg/kg dissolved in 0.9% NaCl, 1 daily injection during 5 days) one month after completion of the behavioral tasks in order to prevent measuring a direct effect of testing on cell proliferation and to analyze basal neurogenesis levels.

#### GFP-retrovirus injections

GFP-encoding retrovirus was produced as previously described^34^. Mice from batch 3 were anesthetized with a mixture of ketamine (100 mg/kg; Imalgene 1000, Merial)/xylazine (10 mg/kg; Rompun, Bayer HealthCare) and received 100 μl of a local anesthetic (Lidocaïne) under the skin covering the skull. They received a unilateral stereotaxic injection of the viral preparation (coordinates from Bregma: AP −2, ML +/−1.8, DV −2.2). Injections (1 μl) were performed using a pulled microcapillary glass tube at a rate of 0.25 µl/min.

#### Immunohistochemistry and stereological analysis

One month after BrdU labeling (batch 1) or GFP injections (batch 3), animals were anesthetized and perfused transcardially with 0.1 M phosphate buffered saline (PBS, pH 7.4), followed by 4% buffered paraformaldehyde (PFA). Brains were collected and post-fixed in PFA at 4 °C for a week. Subsequently, 40 µm-thick coronal sections were cut using a vibratome (Leica) and stored in cryoprotectant medium (30% ethylene glycol, 30% glycerol in KPBS) at −20 °C before staining.

Free-floating sections were processed in a standard immunohistochemical procedure in order to visualize BrdU (1/1000, Accurate OBT0030), doublecortin (DCX; 1:8000; Sigma D9818), Ki67 (1:1000, Novocastra NCL-Ki67P), or GFP (1/8000, Millipore AB3080P)-labeled cells. Briefly, after washing in PBS, sections were treated with methanol and 0.5% H_2_O_2_ for 30 min. Sections were washed again in PBS before incubation with a blocking solution containing 3% normal serum and 0.3% Triton X100 in PBS for 45 min at room temperature. They were then incubated for 48 h at 4 °C with the primary antibodies diluted in the blocking buffer. The following day, sections were incubated with biotin-labeled secondary antibodies diluted in PBS—0.3% Triton X100—1% normal serum, and immunoreactivities were visualized by the biotin–streptavidin technique (ABC kit; Dako) with 3,3′-diaminobenzidine (DAB) as chromogen. The number of immunoreactive (IR) cells throughout the entire granule and subgranular layers of the left DG was estimated using the optical fractionator method^35,36^. The volume of the granular cell layer (GCL) was determined on BrdU-IR stained sections at X400 with the StereoInvestigator software (MicroBrightField, Colchester, VT, USA) and cell density is expressed as number of cells/mm^3^.

For phenotyping newborn cells, double immunofluorescent BrdU-NeuN labeling was performed; floating sections were first treated with 2 N HCl (30 min at 37 °C), incubated for 45 min in PBS containing 5% goat normal serum and 0.3% triton-X-100, followed by 72 h of incubation with a mixture of rat anti-BrdU (1/1000; Accurate OBT0030) and mouse anti-NeuN (1/1000, Millipore MAB377) antibodies in PBS-Triton-X-100. Immunoreactivities were revealed with Alexa 488 goat anti-mouse (1/1000, Invitrogen A11001) and Cy3 goat anti-rat (1/1000, Jackson 112-165-062) secondary antibodies. Sections were mounted on glass slides and coverslipped with polyvinyl alcohol mounting medium with 1,4-diazabicyclo[2.2.2] octane (PVA-DABCO). The percentage of BrdU-labeled cells co-expressing NeuN was determined throughout the DG. For each animal, BrdU-positive cells were randomly selected and analyzed for coexpression with NeuN using a confocal microscope (DMR TCS SP2; Leica Microsystems) and 1 µm interval steps of analysis.

#### Golgi staining

A separate batch of animals (Batch 4) was perfused transcardially with 2% paraformaldehyde and 2.5% glutaraldehyde in 0.1 M PBS, pH 7.4. Coronal vibratome sections for Golgi impregnation (100 µm) were treated with 1% osmium tetroxide in PB for 30 min. They were then placed in 3.5% potassium dichromate overnight, followed by 6 h in 2% silver nitrate solution. The sections were finally dehydrated in graded alcohols, infiltrated in epoxy resin, mounted, and coverslipped on glass slides^37^.

#### Morphometric analysis of GFP-labeled and Golgi-labeled neurons

The overall dendritic tree of GFP-immunoreactive and Golgi dentate granule neurons was measured as previously described^37,38^. Briefly, the morphometric analysis was performed with a ×100 objective using a semiautomatic neuron tracing system (Neurolucida; MicroBrightField, Colchester, VT, USA). Neurons were traced in their entirety, and area of cell body, number of dendritic nodes, and total dendritic length were calculated. To measure the extent of dendritic growth away from the soma and the branching of dendrites at different distances from the soma, a Sholl analysis^39^ was carried out.

#### Statistical analysis

All statistical analyses were performed with Statistica 12.0 software (Statsoft) and results are reported Table 1. Normality was checked with the Shapiro-Wilk normality test. Student t-tests were used for comparing genotypes in anxiety-related and depression-related behavior, as well as in adult neurogenesis; Two-way ANOVAs with genotype and session as main factors were used whenever repeated measures were recorded and followed by a Tukey post-hoc analysis when appropriate. Scores for pain threshold in response to brief ascending foot shocks and for nest building quality were compared by means of Mann–Whitney U-test. In each analysis, a value of *p* < 0.05 was considered significant. All data are presented as mean + SEM.

**Table 1:** Statistics table.

	Variable	Measured effect	*F* (DFn, DFd)	*P* value
Spatial learning and memory				
Water maze variable start	Latency to platform	Genotype effect	*F*_1,20_ = 1.22	*p* = 0.28
		Day effect	*F*_16,320_ = 16.27	***p*** < ***0.0001***
		Genotype × day interaction	*F*_16,320_ = 0.42	*p* = 0.97
	Distance to platform	Genotype effect	*F*_1,20_ = 0.41	*p* = 0.52
		Day effect	*F*_16,320_ = 6.75	***p*** < ***0.0001***
		Genotype × day interaction	*F*_16,320_ = 0.70	*p* = 0.79
Water maze constant start	Latency to platform	Genotype effect	*F*_1,20_ = 2.03	*p* = 0.16
		Day effect	*F*_4,80_ = 9.06	***p*** < ***0.001***
		Genotype × day interaction	*F*_4,80_ = 1.30	*p* = 0.27
	Distance to platform	Genotype effect	*F*_1,20_ = 4.14	*p* = 0.06
		Day effect	*F*_4,80_ = 9.72	***p*** < ***0.001***
		Genotype × day interaction	*F*_4,80_ = 1.97	*p* = 0.11
Water maze novel start	Latency N compared to C5	Genotype effect	*F*_1,20_ = 0.49	*p* = 0.48
		Trial effect	*F*_1,20_ = 0.16	*p* = 0.68
		Genotype × trial interaction	*F*_1,20_ = 0.01	*p* = 0.91
	Latency N compared to C5	Genotype effect	*F*_1,20_ = 0.43	*p* = 0.51
		Trial effect	*F*_1,20_ = 0.16	*p* = 0.69
		Genotype × trial interaction	*F*_1,20_ = 0.000	*p* = 0.99
	Latency trial N/distance trial N	WT vs. S6K1-KO	*t*_20_ = 0.61/*t*_20_ = 0.61	*p* = 0.54/*p* = 0.61
Associative learning				
Contextual fear conditioning	Freezing response	Genotype effect	*F*_1,20_ = 9.01	***p*** = ***0.007***
		Day effect	*F*_2,40_ = 75,45	***p*** < ***0.001***
		Genotype × day interaction	*F*_2,40_ = 4.52	***p*** = ***0.01***
Footshock sensitivity	Flinching/jumping/vocalizing	WT vs. S6K1-KO	*Z* = −0.49/*Z* = −1.34/*Z* = 1.11	*p* = 0.56/*p* = 0.14/*p* = 0.25
Hot plate test		Genotype effect:	*F*_1,20_ = 2.09	*p* = 0.16
		Genotype x temperature effect	*F*_2,40_ = 1.59	*p* = 0.21
Anxiety-like behavior				
Baseline				
Elevated plus maze	OA entries	WT vs. S6K1-KO	*t*_19_ = 2.94	***p*** = ***0.008***
	Time in OA	WT vs. S6K1-KO	*t*_19_ = 3.54	***p*** = ***0.002***
	Distance in OA + CA	WT vs. S6K1-KO	*t*_19_ = −1.55	*p* = 0.13
	OA + CA entries	WT vs. S6K1-KO	*t*_19_ = −1.41	*p* = 0.17
Open field	Distance in corners	WT vs. S6K1-KO	*t*_20_ = −2.80	***p*** = ***0.01***
	Total distance	WT vs. S6K1-KO	*t*_20_ = −1.66	*p* = 0.12
Light/dark emergence task	Exits from cylinder	WT vs. S6K1-KO	*t*_20_ = 1.17	*p* = 0.2
	Total distance	WT vs. S6K1-KO	*t*_20_ = 0.14	*p* = 0.88
Running				
Elevated plus maze	OA entries	WT vs. S6K1-KO	*t*_20_ = 2.92	***p*** = ***0.008***
	Time in OA	WT vs. S6K1-KO	*t*_20_ = 3.13	***p*** = ***0.005***
Open field	Distance in corners	WT vs. S6K1-KO	*t*_20_ = −3.13	***p*** = ***0.005***
Light/dark emergence task	Exits from cylinder	WT vs. S6K1-KO	*t*_20_ = 2.19	***p*** = ***0.04***
Novelty-induced activity drive				
Locomotor response to novelty	Infrared counts	Genotype effect	*F*_1,19_ = 0.02	*p* = 0.89
		Time effect	*F*_5,95_ = 23.10	***p*** < ***0.001***
		Genotype × time interaction	*F*_5,95_ = 0.90	*p* = 0.48
Novel object exploration	Time in target zone	Genotype effect	*F*_1,20_ = 0.17	*p* = 0.69
		Object effect	*F*_1,20_ = 7.47	***p*** = ***0.01***
		Genotype × object interaction	*F*_1,20_ = 0.03	*p* = 0.87
Depression-like behavior				
Nest building	Nest score	WT vs. S6K1-KO	*Z* = 0.240	*p* = 0.81
Splash test	Latency to groom	WT vs. S6K1-KO	*t*_10_ = 2.61	***p*** = ***0.02***
	Frequency grooming	WT vs. S6K1-KO	*t*_10_ = −2.56	***p*** = ***0.02***
Sucrose preference test	Sucrose preference	WT vs. S6K1-KO	*t*_10_ = 0.65	*p* = 0.52
	Sucrose intake	WT vs. S6K1-KO	*t*_10_ = −0.64	*p* = 0.53
Forced swim test	Latency to immobility	WT vs. S6K1-KO	*t*_10_ = −1.99	***p*** = ***0.07***
	Duration of immobility	WT vs. S6K1-KO	*t*_10_ = 2.18	***p*** = ***0.054***
Adult neurogenesis				
Ki67-IR cell number	Whole DG	WT vs. S6K1-KO	*t*_20_ = 4.25	***p*** < ***0.001***
	Left/right/dorsal/ventral	WT vs. S6K1-KO	*t*_20_ = 4.09/*t*_20_ = 3.62/*t*_20_ = 3.94/*t*_20_ = 3.89	***p*** < ***0.001***
BrdU-IR cell number	Whole DG	WT vs. S6K1-KO	*t*_20_ = 3.37	***p*** < ***0.01***
	Left/right/dorsal/ventral	WT vs. S6K1-KO	*t*_20_ = 2.22/*t*_20_ = 4.16/*t*_20_ = 3.26/*t*_20_ = 2.21	***p*** < ***0.01***
DCX-IR cell number	Whole DG	WT vs. S6K1-KO	*t*_19_ = 7.90	***p*** < ***0.001***
	Left/right/dorsal/ventral	WT vs. S6K1-KO	*t*_19_ = 6.28/*t*_19_ = 7.99/*t*_19_ = 7.06/*t*_19_ = 5.95	***p*** < ***0.001***
GFP-IR cells morphology	Cell body area	WT vs. S6K1-KO	*t*_12_ = 0.366	*p* = 0.72
	Number of nodes	WT vs. S6K1-KO	*t*_12_ = 0.012	***p*** = ***0.01***
	Total length	WT vs. S6K1-KO	*t*_12_ = 2.285	***p*** = ***0.04***
	Sholl analysis	Genotype effect	*F*_1,12_ = 5.90	***p*** = ***0.03***
Golgi cells morphology	Cell body area	WT vs. S6K1-KO	*t*_34_ = −0.53	*p* = 0.5
	Number of nodes	WT vs. S6K1-KO	*t*_34_ = 2.11	***p*** = ***0.04***
	Total length	WT vs. S6K1-KO	*t*_34_ = 2.60	***p*** = ***0.01***
	Sholl analysis	Genotype effect	*F*_1,34_ = 5.95	***p*** = ***0.02***

Bold values indicates statistical significant *P* values.

## Results

### Removal of S6K1 specifically alters contextual associative fear memory

**Fig. 2:**
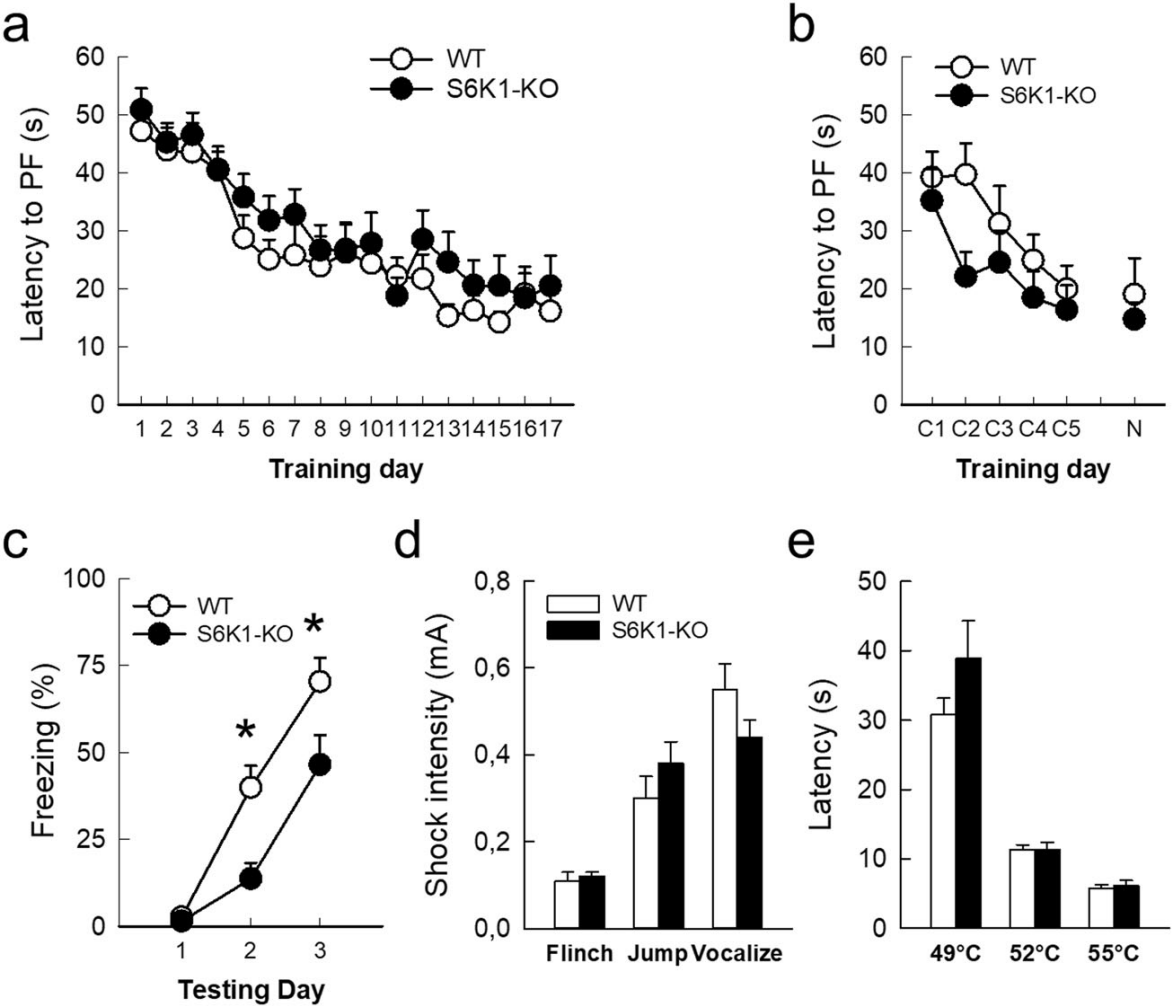
Removal of S6K1 spares spatial navigation but impairs contextual fear memory. **a** Latency to find the platform during reference memory testing (**b**) latency to find the platform location from constant start positions (C1 to C5) and a novel start position (N). **c** Freezing behavior (% total time) in response to a shock-associated context. **d** Threshold to elicit a flinch, jump or vocalize behavior in response to shocks of ascending intensity. **e** Latency to paw licking in response to ascending temperatures in the hot plate test. Data are mean ± SEM. *n* = 11 mice per genotype. *p < 0.05 compared to WT.

We first examined whether removal of S6K1 impairs spatial learning and memory abilities by testing spatial navigation in the water maze. In this task, animals learn the location of a hidden platform using distal cues. It can be solved using multiple strategies in parallel, which requires the integrity of the hippocampus to different degrees. In the first procedure, the platform was maintained hidden (NW quadrant), and the starting point (NE, SW, or SE) was changed at each of the 3 daily trials. In order to find the hidden platform, the animal has to use an allocentric mapping strategy that consists of learning the positional relationships linking the cues (spatial relational memory). This relational representation is needed for using these cues in novel situations (i.e., changing starting position) and is consequently necessary to solve the task. This cognitive ability relies on the integrity of the hippocampus. Under these conditions, mice from both genotypes learned the platform position at a similar rate as seen by the diminution of latency and distance (Fig. 2a, Table 1) necessary to find the platform. In the second procedure, the position of the hidden platform was changed (NW to NE) but the starting point was maintained constant for all trials (SW quadrant). In this case, although the development of a mapping strategy is not prevented, the animal can also learn the position of the platform using egocentric strategies consisting of, for example, the association of an invariant configuration of spatial cues to the escape platform (place learning). Egocentric strategies are very efficient for finding the platform if the starting point is maintained constant but fail to sustain the behavior if the starting point is suddenly changed. Under these conditions, mice of the 2 genotypes did not differ in the daily evolution of latency and distance to find the platform during the constant-start learning phase (Fig. 2b C1 to C5, Table 1). When they were released from a new starting point at the end of the learning phase, all mice were able to find the platform, and performances did not differ between genotypes (Fig. 2b trial N, Table 1). Taken together results of the 2 procedures confirm that mice of both genotypes present similar abilities in spatial memory and are able to develop an efficient relational strategy.

We then tested whether removal of S6K1 could alter the ability of mice to form and remember an association in the contextual fear conditioning task. If learning occurs, further exposure of an animal to the conditioning environment where it received an electric foot-shock elicits a freezing fear response. Mice (batch 1) received a single foot-shock each day during 3 days and their freezing response to the context-associated shock was recorded every day before shock exposure. Although both WT and S6K1-KO mice displayed an increased freezing across days, the latter reached much lower levels of freezing than WT (Fig. 2c, Table 1). This difference was visible only on day 2 and day 3 (Tukey post-hoc test: day 1 *p* = 0.99; day 2 *p* = 0.01; day 3 *p* = 0.03), indicating that the reduced freezing of S6K1-KO mice is not due to baseline differences but it is linked to a specific impairment in their ability to acquire a contextual associative fear memory. We further controlled that these differences were not due to an alteration of nociceptive sensory perception by measuring pain threshold in response to brief ascending foot-shocks or temperature setpoints. For both tests, the two groups did not differ (Fig. 2d, e, Table 1), confirming that the decreased freezing observed in S6K1-KO mice is not linked to a lowered pain perception.

### Removal of S6K1 increases anxiety-like but not depression-like behavior

**Fig. 3:**
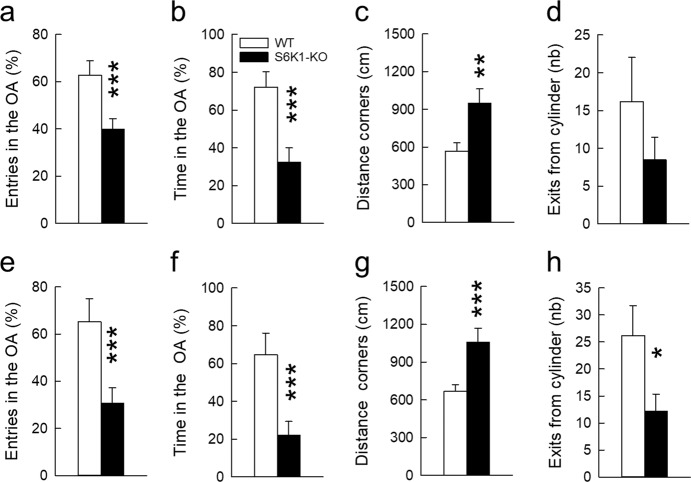
Removal of S6K1 increases anxiety-related behaviors. Anxiety-like responses were measured in the elevated plus maze (**a, b, e, f**), in the open-field (**c, g**) and in the light/dark emergence task (**d, h**) under baseline conditions (top) or after cage enrichment with a running wheel (bottom). Data are mean ± SEM, *n* = 11 per genotype and per test except for the elevated plus maze under baseline conditions where one S6K1-KO mouse was removed as it fell from the maze. *p < 0.05, **p < 0.01, and ***p < 0.001 compared to the WT.

Anxiety-related behavior in rodents is mostly studied by measuring avoidance responses to potentially threatening situations, such as unfamiliar open environments. We first tested anxiety in the elevated plus maze (EPM) composed of two closed arms and two open arms, the latter constituting the threatening areas. Avoidance for these threatening areas was largely increased in S6K1 mutant mice, which visited less and spent less time in the open arms (Fig. 3a, b, Table 1). When exposed to a bright open-field (OF), again the behavior of the two groups was different as the distance traveled in the safest areas of the open field, the corners, was higher in mutant compared to control mice (Fig. 3c, Table 1). Finally, mice were tested in the light/dark emergence task, a free exploration task in which animals can explore a brightly lit OF or retreat into a dark and reassuring cylinder. The number of exits from the cylinder, considered as an index of a lowered anxiety, was slightly decreased in the mutant mice, albeit this effect did not reach statistical significance (Fig. 3d, Table 1).

**Fig. 4:**
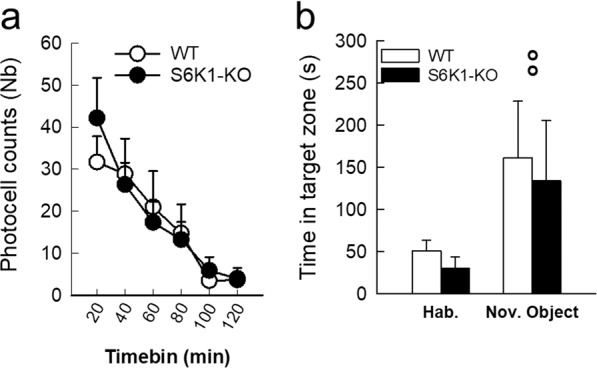
Removal of S6K1 does not alter activity drive in response to novelty. Locomotor activity was recorded in response to a novel environment (**a**) and exploration was recorded in presence of a novel object (**b**). Data are mean ± SEM with *n* = 11 per group, except for WT *n* = 10 in response to novelty as one activity cage was deficient. °°*p* < 0.01 compared to the habituation phase.

We verified that changes in activity/exploratory drive did not account for these phenotypic differences first by analyzing the exploratory tendency of mice in the different tests, then by measuring their activity drive in response to novelty in non-threatening situations. No differences in activity could be evidenced in the EPM (Distance traveled in both open and closed arms: WT: 7.95 + 0.9 m vs. S6K1-KO: 10.31 + 1.2 m; Total number of entries in both open and closed arms: WT: 17.09 + 2.0, S6K1-KO: 21.5 + 2.3; Table 1), the OF (total distance traveled:WT: 21.9 ± 1.5 m vs. S6K1-KO: 31.8 ± 5.7 m; Table 1), or the light/dark test (total distance traveled: WT: 23.9 ± 3.6 m vs. S6K1-KO: 22.9 ± 5.5 m; Table 1). To test the activity drive in response to novelty, mice were tested for novelty-induced locomotor activity and novel object-induced exploratory activity in non-threatening environments. Both groups showed a similar decrease over time in locomotor activity as the context lost its novelty (Fig. 4a, Table 1) and similarly explored the novel object, as shown by the increase in the time spent in the target zone when the object was present (Fig. 4b, Table 1). These data indicate that the increased avoidance of threatening areas observed in S6K1-KO mice in the EPM, OF and light/dark tests is not linked to an impairment in exploratory drive but likely reflects increased anxiety.

Then we asked whether anxiety-related behavior was stable and maintained when home cages were enriched by adding a running wheel, as running might bear anxiolytic potential^40^. Mice did not differ in their average daily use of the wheel (WT = 1182 + 503 wheel revolutions; S6K1-KO = 1142 + 470 wheel revolutions; *t*_20_ = 0.059, *p* = 0.95). After 21 days of exposure to this new housing condition, anxiety-like responses were measured as previously done in the EPM (Fig. 3e, f), the OF (Fig. 3g) and the light/dark emergence task (Fig. 3h). We found that the anxious phenotype was maintained and even more pronounced as differences between groups reached significance in the light/dark test (Table 1). Altogether these data indicate that anxiety-like behavior is an enduring feature of S6K1 mutant mice.

**Fig. 5:**
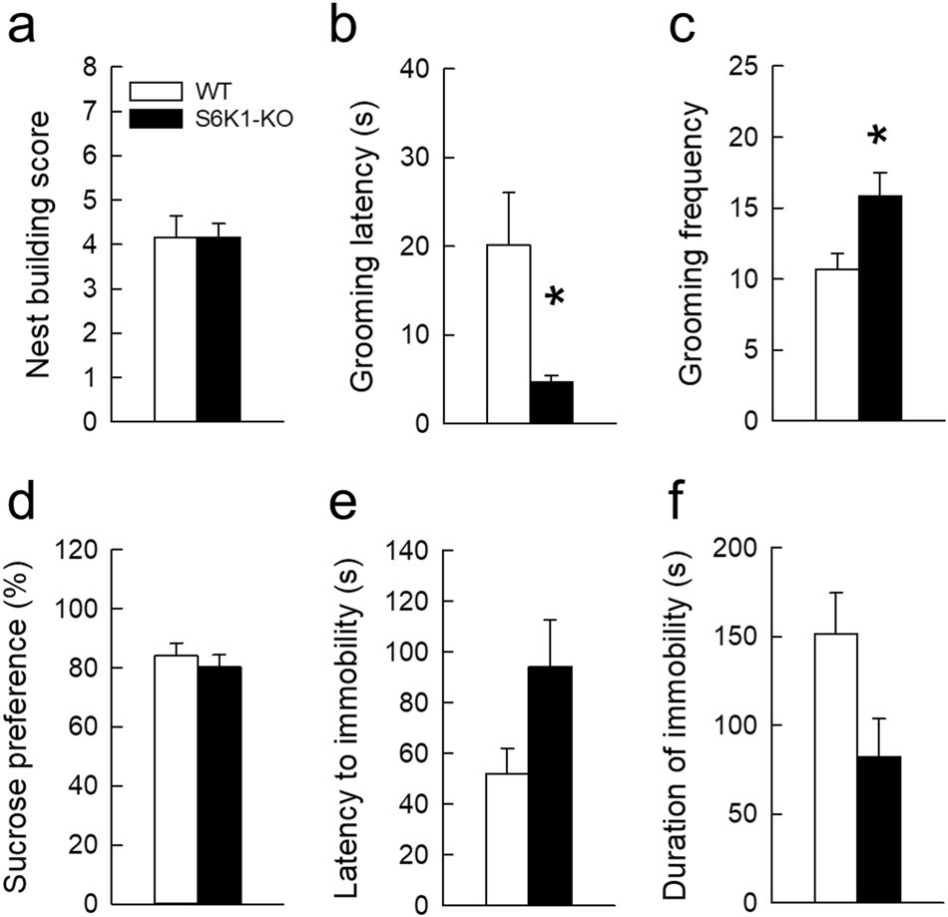
Removal of S6K1 does not increase depression-like responses. Motivation was evaluated in the nest building (**a**) and the sucrose splash test (**b**, **c**); anhedonia was measured in the sucrose preference test (**d**); and resignation was evaluated in the forced swim test (**e**, **f**). Data are mean ± SEM. *n* = 6 per genotype. *p < 0.05 compared to WT.

In the last series of experiments, the impact of removing S6K1 was examined on depression-related behavior using readouts for lack of motivation (*nest building*, Fig. 5a, *grooming in the splash test*^41^, Fig. 5b, c), anhedonia (*sucrose preference*, Fig. 5d), or resignation (*forced swim test*, Fig. 5e, f).

Nest quality was not different between groups (Fig. 5a, Table 1) indicating that spontaneous motivation is spared following S6K1 deletion. Animals’ motivation toward self-centered activities was evaluated by measuring grooming behavior. Typically, frequency and extent of grooming behavior is impaired in rodent models of depression leading to a degradation of coat states. Given that the coat states did not differ between the two groups of animals, we stimulated grooming behavior by splashing the back of the mice with a high viscosity sucrose solution. Opposite to what was expected, S6K1-KO mice developed an increased grooming behavior as shown by a decreased latency (Fig. 5b, Table 1) and an increased frequency of grooming (Fig. 5c, Table 1). In the sucrose preference test, in which a decrease in sucrose consumption is considered as an index of anhedonia, we found no differences between groups in both sucrose preference (Fig. 5, Table 1) and intake (WT: 11.4 ± 1.4 ml/g body weight; S6K1-KO: 12.9 ± 1.9 ml/g body weight, Table 1). In the forced swim test, although a strong tendency to increased active coping reflected by an increased latency to immobility (Fig. 5e) and a deceased immobility time during the last 4 min of the test (Fig. 5f) was recorded in S6K1-KO mice, this effect did not reach statistical significance (Table 1).

When analyzed together, this last dataset clearly indicates that S6K1-KO mice do not exhibit any consistent sign of anhedonia or “behavioral despair”. The analysis of each individual test wherein WT and S6K1-KO mice differ may suggest increased motivation, but confounding factors cannot be excluded. Indeed, the excessive self-grooming displayed by S6K1-KO mice in the splash test could be linked to an increased propensity to obsessive-like response, but to the best of our knowledge, this type of behavior has never been tested in this model. As for differences in swimming behavior in the FST, buoyancy issues could be at play. Indeed, as previously reported^28^ S6K1-KO mice are smaller and we cannot exclude that decreased floating capabilities due to their lower body mass (WT *m* = 43.55 + 1.4 g; S6K1-KO *m* = 26.38 + 1.7 g; *t*_10_ = 7.52, *p* < 0.001) translates into an increased swimming propensity in order to maintain flotation.

### Removal of S6K1 decreases adult hippocampal neurogenesis

**Fig. 6:**
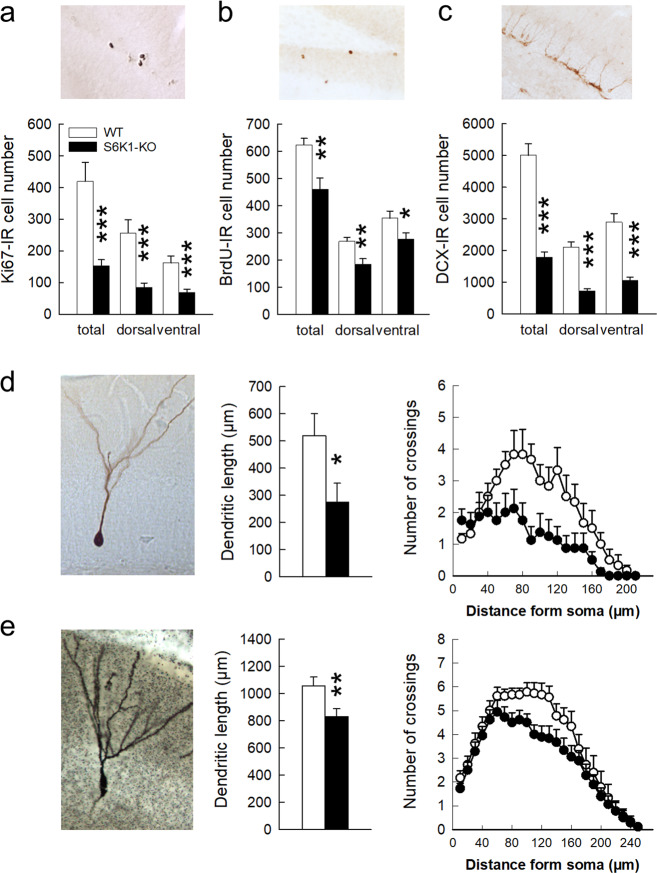
Removal of S6K1 decreases adult hippocampal neurogenesis and alters dentate granule cells morphology. **a** Cell proliferation, (**b**) Cell survival, (**c**) Neurogenesis, (**d**) Dendritic arborization of 4 weeks old adult born dentate neurons, (**e**) Dendritic arborization of dentate neurons impregnated with Golgi (*n* = 3 per group). Data are mean ± SEM with *n* = 11 per genotype for **a**, **b**, **c**; *n* = 6 WT and 8 S6K1-KO for **d**; *n* = 18 neurons per genotype for **e**. *p < 0.05, **p < 0.01, and ***p < 0.001 compared to WT.

Cell proliferation, examined using Ki67, was strongly decreased in S6K1-KO mice (Fig. 6a, Table 1). This decrease was observed in both hemispheres and concerned both the dorsal and ventral parts of the dentate gyrus. This translated into a decreased number of 1-month old surviving BrdU-labeled cells (Fig. 6b, Table 1) that was observed in both the left and right dentate gyrus, and concerned both the dorsal and ventral parts, as well as a decrease in the number of doublecortin-positive immature neurons (Fig. 6c, Table 1) that was again observed in all sub-regions. As mentioned previously, S6K1-KO mice are smaller than their WT counterparts so we verified that the decreased BrdU-IR cell number was not simply related to a decreased hippocampal volume. Despite their difference in body weight and size, the volume of the DG in S6K1-KO mice did not differ from that of WT mice (WT = 0.80 + 0.01 mm^3^ vs. S6K1-KO = 0.85 + 0.02 mm^3^; *t*_20_ = −2.06, *p* = ns) and as a result the density of BrdU cells was strongly decreased in mice deleted for S6K1 (WT = 779 + 31 cells/mm^3^ vs. S6K1-KO = 542 + 48 cells/mm^3^; *t*_20_ = 4.08, *p* < 0.001). Before concluding that this decreased BrdU cell number translated into a decreased production of newborn neurons, we also verified whether neuronal differentiation was modified in S6K1-KO mice and found no differences in the percentage of BrdU-immunoreactive cells that also express the neuronal marker NeuN (WT = 88.1 + 1.9% vs. S6K1-KO = 86.7 + 1.1%; *t*_10_ = 0.60, *p* = ns).

Altogether, this indicates that removal of S6K1 decreases cell proliferation and alters adult neurogenesis. As the relevance of adult neurogenesis also relies on the synaptic integration of newborn cells, we checked the impact of S6K1 removal on the morphology of 4-wks old newborn neurons (Table 1). To this end, we used a GFP-encoding retrovirus that infects only dividing cells and allows cytoplasmic expression of GFP, thus providing a tool for dendritic analysis. Although the cell body area of newborn cells was not affected by the deletion of S6K1 (WT = 98.28 + 6.9 µm^2^, S6K1-KO = 94.78 + 6.4 µm^2^), all other parameters pointed to an atrophy of adult-born granule cells in S6K1-KO mice, which displayed less nodes (WT = 6.16 + 0.5, S6K1-KO = 2.75 + 0.9), shorter length (Fig. 6d), and decreased complexity of dendrites (Fig. 6d). Finally, in order to test whether this dendritic atrophy is restricted to adult-born cells or affects the entire population of granule neurons, we evaluated the morphology of neurons impregnated with Golgi on a separate set of animals (Table 1). As for newborn neurons, we found an atrophy (number of nodes WT = 9.11 + 0.6, S6K1-KO = 7.38 + 0.5; total length, Fig. 6e) and a decreased complexity of dentate granule neurons in mutant mice (Fig. 6e) without modifications of cell body area (WT = 126.1 ± 8.9 µm², S6K1-KO = 133.2 ± 9.8 µm²).

## Discussion

There is a high comorbidity between neurodegenerative disorders, in which mTOR inhibitors are used as therapeutic approaches, cognitive defects and emotional dysregulation, such as anxiety and depression. Moreover, the mTOR pathway itself has been involved in learning and memory^7^ and recent studies have highlighted the involvement of mTORC1 signaling in stress-associated disorders, and in particular in depression^42^. The involvement of this pathway in anxiety has however remained elusive. Here we show for the first time that S6K1, a downstream target of mTORC1, mediates anxiety-like behaviors. Using a well-established transgenic mouse model, we confirm that S6K1 has a major impact on shock-induced contextual associative fear memory, and report unequivocally that removal of S6K1 increases anxiety-like behavior. These alterations are associated with a defect in adult neurogenesis and a global atrophy of dentate neurons.

Studies that have so far investigated the role of mTORC1 signaling in regulating emotional states are sparse, and reports concerning both anxiety-like and depression-like behavioral effects are controversial. In line with our own results on anxiety, it has been shown that exposure to a mild stress decreases hippocampal mTORC1 signaling and increases anxiety-like behavior^43^. On the same line, anxiolytic effects of fast-acting antidepressant drugs, such as YY-21, require activation of mTORC1 signaling in the medial prefrontal cortex (mPFC)^44^ and exercise, which can reduce the incidence of anxiety^45^, increases mTOR activity in the hippocampus and mPFC in rats^46^. Although this last dataset supports our findings, it was also reported that viral-mediated increased expression of S6K1 in the mPFC does not influence anxiety-like behaviors^47^. Furthermore, no differences in anxiety levels were found when testing the same genetic model of S6K1 deletion in an open-field^30^. In addition, pharmacological manipulation of the mTOR pathway with prescribed inhibitors also results in anxiogenic or anxiolytic effects, depending on the dose, route of administration, age of the subjects, and animal model used, as well as on possible preexisting neuropsychiatric predispositions or experimentally-induced neurological damage. For instance, chronic rapamycin treatment improved anxiety-like behaviors throughout lifespan in mice^17^, whereas treatment of male mice with the rapamycin analog everolimus induces anxiety-like behavior^11^. Consistent with this latter result, chronic treatment with rapamycin had anxiogenic effects in male rats^10,48^, as well as in a mouse model of Fragile X Syndrome, a neurodevelopmental disorder characterized by an upregulated mTORC1 signaling^49^. Finally, one study reported that rapamycin treatment in rats increased anxiety in a battery of tests without modifying phospho-S6K1 protein levels^10^. This observation led the authors to suggest that anxiety-related behavior after treatment with mTOR inhibitors could not directly be attributed to mTOR-dependent mechanisms. Our own data, directly testing the involvement of S6K1, contradict this hypothesis and strongly suggest that anxiety induced by mTOR inhibitors can indeed be linked to an inhibition of the mTORC1 pathway. Altogether, the evidence currently available in the literature clearly point to a need to further investigate when and under which circumstances manipulation of mTORC1 signaling may differentially impact anxiety. More specifically, because one of the main differences between our model and for instance chronic treatment of adult animals with mTOR inhibitors or virally-mediated alterations in mTORC1 pathway, is the developmental period and the length of time during which S6K1 activity is altered, an interesting first step would be to determine whether there is a critical time window for the involvement of S6K1 in driving anxiety-like behavior. The availability of cre inducible models in which S6K1 expression can be altered either during the early phases of development or in adulthood should help define this critical time-window, which ultimately will allow dissecting the underlying mechanisms. Furthermore, because anxiety consists of a complex response system encompassing cognitive, affective, physiological, and behavioral components^50^, a more refined behavioral analysis associating approach-avoidance tests such as the ones we used with other measures of defensive behavior could be engaged to better characterize the behavioral impairment induced by S6K1 failure.

Although it was even less thoroughly tested, the involvement of mTORC1 in depression-like behavior is similarly controversial. In our study, genetically blocking mTORC1 signaling does not induce depressive-like symptoms, which contrasts with previous reports indicating that reducing mTORC1 activity through 3-week treatment with everolimus^11^ or virally-mediated suppression of S6K1 activity in the mPFC of adult mice^47^ increases depressive-like behaviors. Alternatively, subchronic/chronic rapamycin treatment in both mice and rats was found to exert antidepressive-like effects^16,17,51^. Interestingly, a recent study reported that depression-like and anxiety-like behaviors exhibited by mice in a Parkinson Disease (PD) model are eliminated by rapamycin, but not by selective blockade of the mTORC1 downstream target, S6K1^52^. Keeping in mind that these results were obtained in a PD-animal model, they can partly explain discrepancies in the existing data, and strongly corroborate the fact that inhibition of S6K1 does not recapitulate rapamycin actions. They also highlight the importance of gathering additional data on the consequences of manipulating downstream molecular targets of mTORC1 to isolate potential candidates for medicating psychiatric symptoms both in baseline conditions and for comorbidities in neurological diseases where mTOR malfunctioning is manifest^10^.

In line with previous reports highlighting a role for mTORC1 signaling in both associative and non-associative fear memories^53^, our data also show that constitutive deletion of S6K1 causes a deficit in shock-induced contextual associative fear memory, while spatial navigation is spared. This dataset is consistent with reports that S6K1 is required for acquisition and consolidation of normal contextual fear memory but not necessary for spatial navigation using the same animal model^30^. The inability to acquire a shock-induced contextual associative fear was not due to an alteration of nociceptive sensory perception. Thus, we propose that an enhanced emotional reactivity linked to an anxious phenotype could be at the origin of the associative fear deficits. Supporting this view, rapamycin administration blocks predator stress-induced associative fear memory^53^, as well as shock-induced inhibitory avoidance^3^.

In our studies, the increased emotional reactivity of S6K1-KO mice was associated with a reduction of adult neurogenesis. Cell proliferation was reduced in the DG, and as a consequence, the number of surviving cells and the number of immature neurons expressing DCX were also decreased. The complexity of dendritic arbors of both adult-born and developmentally-born granule neurons was also altered as revealed by the diminution of dendritic length and complexity. Here again controversial data have been collected regarding mTOR and adult neurogenesis but the overall majority of studies seem to reach a consensus indicating that inhibition of the mTOR pathway decreases progenitor pools and neurogenesis^54–56^, which agrees with our own results. Only one study reported increased neurogenesis after chronic administration of everolimus^11^, and another one reported no effect of everolimus treatment on cell proliferation^18^. It should be noted that in this last study no behavioral consequences were observed after treatment and it is possible that their regimen of administration was subthreshold. Our data are also in agreement with work from Dwyer et al. who reported increased dendritic branching of cortical neurons after transfection with a constitutively active form of S6K1^47^. Although the impact of inhibiting the activity of S6K1 was not tested, this is consistent with our own data and indicates that the relationships between S6K1 and neuron morphology extend to different structures. Although we did not directly test this hypothesis, it is highly conceivable that this alteration in adult neurogenesis could mediate the increased anxiety observed in S6K1-KO. Indeed, hippocampal adult neurogenesis has emerged over the last decades as a central mechanism contributing to hippocampal function and although controversies were recently raised regarding its existence in humans^57–59^ much evidence point to methodological concerns to explain discrepancies in reports of neurogenesis in the human brain^60,61^, and the currently accepted view is that neurogenesis does occur in the human brain throughout lifespan. Although its functional role cannot yet be deciphered in humans, literature from animal models has consistently reported functions of adult neurogenesis in complex hippocampal-dependent memory processes, as well as in the regulation of emotional behaviors^62–67^. More specifically in regard with the anxiety-like phenotype exhibited by S6K1-KO mice, we and others have reported that disruption of adult neurogenesis by silencing, removing, or reducing adult-born neurons, increases avoidance responses and defensive reactions, thus favoring anxiety-like behaviors^68–70^. Interestingly, such disruption of adult neurogenesis is not accompanied by depression-like symptoms^68,71^, which is consistent with the absence of a depressive-like phenotype in mice deficient for S6K1. Finally, when considering the involvement of adult neurogenesis in spatial learning and memory^36,66,72^ the lack of behavioral deficits of S6K1-KO mice in the Morris water maze may appear difficult to reconcile with their decreased neurogenesis. However, it has recently emerged that neurogenesis is particularly important for learning when cognitive demand is high and when there is a high possibility for interference between memories; consistently, disruption of neurogenesis usually spares learning in spatial tasks when no overlapping representations are involved^62,72^. It is thus possible that the paradigm used in our study was not stringent enough to reveal subtle deficits in memory processes. Among the alternative mechanisms that could be at play to sustain the behavioral deficits of S6K1-KO mice, altered synaptic plasticity could be a good candidate as it has been linked with learning and memory capabilities and with expression of S6K1^73^. In accordance with this hypothesis, increased protein levels of SAPAP3, a post-synaptic scaffolding protein associated with PSD-95, and lack of phospho FMRP, a repressive RNA binding protein target of S6K1, were reported in hippocampal lysates of S6K1-KO mice^74^. Because FMRP phosphorylation is involved in the signaling cascade leading to mGluR-induced protein synthesis dependent synaptic plasticity, this indicates that lack of S6K1 activity could affect levels of phospho FMRP and thus mGluR dependent synaptic plasticity. However, using the same mouse model as ours, Antion et al. could not evidence any involvement of S6K1 in protein synthesis-dependent synaptic plasticity as late phase LTP and mGluR-dependent LTD, which both depend on protein synthesis, were not modified in S6K1-KO mice^30,75^. Interestingly, the behavioral and neurobiological profile induced by S6K1 deletion is highly reminiscent of a stress-induced phenotype (e.g., increased anxiety (for review see^76^), decreased adult neurogenesis^65^, neuronal atrophy^77^), suggesting that alterations in the HPA axis activity may be involved. Data on the consequences of mTOR pathway inhibition on HPA axis activity are sparse. Nevertheless, one study reported that basal plasmatic levels of corticosterone did not differ between WT and S6K1-KO mice^22^ indicating that the outcomes of S6K1 deletion may not involve elevated stress levels. However, because stress and GR activation were found to alter synaptic plasticity via mTOR pathway dependent mechanism^78^, together with the facts that the mTOR/p70S6K/S6 pathway is responsive to stress^79^ and that deletion of S6K1 leads to a stress-like phenotype (our data) it is tempting to hypothesize that the reported stress effects may be mediated by an mTORC1-dependent mechanism relying on S6K1, a hypothesis that requires further testing and experimental demonstration. Keeping in mind that our genetic model constitutively lacks the expression and activity of S6K1 in all cell types and that the resulting phenotype might be related to neurodevelopmental alterations, our data point toward an important role for mTORC1 signaling, and more specifically S6K1 activity, in the regulation of anxiety-related behavior. As highlighted previously in the discussion, discrepant results have been obtained in the literature regarding the behavioral/neurobiological consequences of manipulating mTORC1 pathway using either global knockout mice, more specific transgenic mice or anti-mTORC1 treatments generally administrated in adulthood. Apart from the diversity of models and experimental conditions under which this pathway is studied, the fact itself that the pathway has pleiotropic cellular effects (spanning from the regulation of mitochondrial function to lipid and protein metabolism) and that it is present in every cell type may clearly explain the different outcomes. This emphasizes the need for more studies on this pathway. In particular, the availability of refined genetic models targeting either upstream regulators of mTORC1 signaling such as Tsc1 or Tsc2 specifically in neural progenitor or astroglial cells, or downstream targets of mTORC1 such as S6K1, S6K2, or members of the eIF4E-binding proteins, coupled with cre or dox on/off driver tools and optico-genetic switches should permit much better spatiotemporal resolution of mTOR signaling and dissection of the molecular players^7^. In a translational perspective, such refinement should allow developing new pharmacological tools that would target specific players of the mTOR pathway with less side effects. In this respect, the major outcome of our study is to open new research avenues to evaluate whether pharmacological manipulation favoring S6K1 activity may favorably impact anxiety disorders.
